# Evaluating toolkits for inclusivity, diversity, equity, and accessibility in clinical trials: a scoping review protocol

**DOI:** 10.3389/fpubh.2026.1824000

**Published:** 2026-06-22

**Authors:** Syamala Buragadda, Eva Friedman, Marie-Annick Clavel, Stuart G. Nicholls, Alison Farrell, Léa-Mai Simard, Mélysiane Marcotte, Manasvi Ganti, Louise Pilote

**Affiliations:** 1 IDEA Committee, Accelerating Clinical Trials Consortium, McGill University Health Center Research Institute, Montreal, QC, Canada; 2 Population Health Research Institute, Accelerating Clinical Trials Consortium, Hamilton, ON, Canada; 3 Institut Universitaire de Cardiologie et de Pneumologie de Québec - Université Laval, Quebec City, QC, Canada; 4 Ottawa Hospital Research Institute, Centre for Practice-Changing Research, General Campus, Ottawa, ON, Canada; 5 Health Sciences Library, Faculty of Medicine, Memorial University of Newfoundland and Labrador, St. John's, NL, Canada; 6 Department of Medicine, McGill University, Montreal, QC, Canada; 7 Faculty of Nursing, Memorial University of Newfoundland, St. John's, NL, Canada; 8 Department of Medicine, McGill University Health Center Research Institute, McGill University, Montreal, QC, Canada

**Keywords:** accessibility, clinical trials, inclusive clinical trials, scoping review, toolkits

## Abstract

**Background:**

Many toolkits have been developed globally to help researchers design and conduct inclusive clinical trials, but their usability for Canada’s diverse population remains unclear. This scoping review aims to evaluate existing toolkits and assess their appropriateness in supporting inclusive clinical trials in Canada.

**Methods:**

We will conduct a comprehensive search of peer-reviewed and grey literature from April to August 2025 across multiple databases, including MEDLINE, EMBASE, CINAHL, PsycINFO, Cochrane, Scopus, and Web of Science. Search terms will combine inclusion, diversity, equity, and accessibility with terms for toolkits and clinical trials. Two independent reviewers will screen titles and abstracts using predefined inclusion and exclusion criteria, and full-text reviews will be conducted for eligible studies. Data will be extracted using a standardized form, focusing on toolkit content. We will summarize findings in a matrix and will present the extracted data in a tabular format, allowing easy comparison across toolkits.

**Discussion:**

This scoping review aims to assess the relevance and usability of existing toolkits that support inclusive clinical trials, with a specific focus on their applicability to the clinical trials context. Given Canada’s diverse population, vast geography, and low participation rates among underrepresented groups, it is essential to determine whether current global resources meet local needs. The findings will help identify content gaps and inform the development of a tailored IDEA toolkit to support equitable and representative clinical research across Canada.

**Ethics and dissemination:**

The protocol was registered on the Open Science framework. The findings will be presented at relevant conferences, and the manuscript will be submitted to peer-reviewed journals.

**Clinical trial registration:**

https://doi.org/10.17605/OSF.IO/UDQBJ.

## Introduction

The foundation principles of clinical research include Inclusivity, Diversity, Equity and Accessibility (IDEA). These principles aim to address underrepresentation and disparities in clinical trials. Historically, certain groups, such as racial and ethnic minorities, women, and individuals from lower socioeconomic backgrounds, have been underrepresented in clinical trials (1, 2). This underrepresentation limits the generalizability and applicability of trial findings to diverse populations (3, 4). It may also contribute to inequitable healthcare interventions (5, 6) and widen the existing health disparities (7).

In response to the growing need for more inclusive clinical trials, IDEA focused toolkits have been developed to guide researchers, trialists, clinical trial units, and research organizations in designing trials and conducting trials that are more representative of diverse populations (12). These toolkits may include guidance documents, frameworks, checklists, templates, training modules, implementation tools, and other practical resources. However, the scope, content, and usability and evidence base of these toolkits vary across jurisdictions and settings. As a result, it remains unclear whether existing are appropriate for the Canadian clinical trial context, where considerations such as indigenous populations, bilingualism, multicultural communities, rural and remote populations, accessibility needs, and regulatory requirements are particularly important (8).

In this protocol, the acronym IDEA refers specifically to Inclusivity, Diversity, Equity, and Accessibility as applied to the design, conduct, analysis, and dissemination of clinical trials. This use of IDEA is distinct from the International Development Ethics Association, which is a separate scholarly organization with a different disciplinary focus, purpose, and mandate. Throughout this manuscript, IDEA is used only in relation to inclusive and equitable clinical trial practices.

A key focus of this review will be to assess how existing toolkits support inclusivity, diversity, equity, and accessibility across the clinical trial lifecycle. This includes examining whether toolkits address recruitment and retention of underrepresented groups, equitable trial access, accessible trial materials and processes, culturally appropriate approaches, disaggregated analysis, and dissemination of findings to relevant communities. By systematically examining existing toolkits, this review aims to identify best practices, highlight gaps, and provide recommendations for adapting or developing toolkits to support inclusive clinical trials in Canada.

## Methods

The primary objective of this scoping review is to identify describe, and evaluate existing toolkits designed to support IDEA in clinical trials. t. Specifically, this review will examine the content, target populations, intended users, practical components, usability features, evidence base, and applicability of existing IDEA-focused toolkits to the Canadian clinical trial context. The review will also identify gaps and opportunities to inform the development or adaptation of future toolkits for inclusive clinical trials in Canada.

This scoping review will be conducted and reported using the PRISMA-ScR (Preferred Reporting Items for Systematic Reviews and Meta-Analyses Extension for Scoping Reviews) guidelines (9). The protocol was registered on the Open Science framework (OSF) (10) and can be accessed at https://doi.org/10.17605/OSF.IO/UDQBJ.

### Research question framework

This scoping review will be guided by the Population/Participants–Concept–Context framework.

The primary research question is:

What toolkits, resources, frameworks, checklists, or guidance documents exist to support Inclusivity, Diversity, Equity, and Accessibility in clinical trials, and how applicable are these resources to the Canadian clinical trial context?

The Population/Participants include researchers, trialists, clinical trial units, research coordinators, healthcare professionals, policymakers, research organizations, patient partners, and other stakeholders or interest holders involved in the design, conduct, analysis, or dissemination of clinical trials.

The Concept is IDEA-focused toolkits or practical resources designed to support inclusive clinical trials. These may include structured guidance documents, frameworks, checklists, templates, training modules, assessment tools, implementation guides, or other practical resources intended to improve IDEA in clinical trial processes.

The Context is clinical trials, with a specific focus on the applicability of identified toolkits to the Canadian clinical trial environment. This includes considerations related to Indigenous population, bilingualism, multicultural communities, rural and remote populations, people with disabilities, Canadian regulatory requirements, and equitable access to trial participation.

### Search strategy

We will conduct a comprehensive search of both peer-reviewed articles and grey literature. The following databases will be searched: MEDLINE (Ovid), EMBASE,^1^ CINAHL (Ebsco), PsycINFO (Ebsco), Cochrane Library, Scopus, and Web of Science Core Collection.

Grey literature sources will include Google Scholar, institutional websites, governmental websites, clinical trial registry websites, and websites of relevant research, funding, and health organizations. These may include, but are not limited to, the Multi-Regional Clinical Trials Center, National Institutes of Health, World Health Organization, ClinicalTrials.gov, International Clinical Trials Registry Platform, Canadian Institutes of Health Research, Health Canada, Statistics Canada, and relevant national and international research organizations.

Search terms will include combinations of terms related to:

1. inclusivity, diversity, equity, accessibility, underrepresented populations, marginalized populations, and underserved groups;
2. toolkits, frameworks, guidance documents, checklists, tools, templates, and training resources; and
3. clinical trials and clinical research.

The search strategy will be developed iteratively with librarian expertise. Because toolkit-related terminology is broad and inconsistently used, the search will intentionally include a wide range of terms to maximize capture of relevant resources. Specificity will be improved during title and abstract screening and full-text review using predefined eligibility criteria. Reference lists of relevant articles and resources will also be reviewed to identify additional sources.

The full search strategy for Medline (Ovid) is included in Appendix 1.

All records will be exported to Covidence systematic review software, (Veritas Health Innovation, Melbourne, Australia. Available at www.covidence.org), where they will be deduplicated before the screening.

#### Operational definition of a toolkit

For this review, an IDEA toolkit will be defined as a structured, practical resource designed to help researchers, trialists, clinical trial units, research organizations, or other stakeholders implement IDEA principles in one or more phases of clinical trial design, conduct, analysis, or dissemination (11).

To be considered a toolkit, a resource must include at least one practical or actionable component intended to support implementation. These components may include checklists, templates, assessment tools, implementation steps, training modules, decision aids, examples, case studies, recruitment or retention strategies, evaluation tools, or structured guidance for applying IDEA principles in clinical trials.

Resources will be eligible if they address at least one IDEA domain:

Inclusivity: Strategies to support meaningful participation of historically underrepresented or underserved populations in clinical trials.
Diversity: Approaches to improve representativeness across age, sex, gender, race, ethnicity, socioeconomic status, geography, disability, health status, and other relevant characteristics.
Equity: Strategies to identify and address structural, social, economic, geographic, cultural, or healthcare-related barriers to trial participation.
Accessibility: Approaches to improve physical, digital, linguistic, cognitive, cultural, or logistical access to clinical trials.

The review will consider whether toolkits address populations that have historically been underrepresented in clinical trials, including women, Indigenous population, racialized communities, immigrants and refugees, rural and remote populations, people with disabilities, older adults, sexual and gender minorities, people with lower socioeconomic status, and other underserved groups.

General editorials, opinion pieces, commentaries, conceptual papers, or frameworks that do not include practical implementation tools or actionable resources will not be considered toolkits for the purpose of this review.

### Eligibility criteria for the studies to be included

Resources will be included if they meet the following criteria:

1. Focus on promoting Inclusivity, Diversity, Equity, and/or Accessibility in clinical trials or clinical research involving trial-related processes.
2. Include practical toolkit content, such as checklists, templates, guidance documents, frameworks, training materials, implementation steps, assessment tools, or other actionable resources.
3. Address at least one phase of the clinical trial lifecycle, including trial design, recruitment, consent, retention, trial conduct, data analysis, interpretation, dissemination, or implementation.
4. Are available as peer-reviewed publications, public reports, organizational documents, institutional resources, or grey literature.
5. Are published in English or French.

Resources will be excluded if they:

1. Are not specific to clinical trials or clinical research.
2. Discuss IDEA principles only conceptually without providing practical or actionable toolkit content.
3. Are editorials, commentaries, opinion pieces, or narrative discussions without toolkit components.
4. Are not available in English or French.
5. Are not publicly accessible or cannot be retrieved through institutional access, public websites, or author/organization contact.

The review will be limited to English and French resources because these are Canada’s two official languages and are the languages in which the review team can reliably screen, extract, and interpret toolkit content. Given the Canadian focus of this review, English and French resources are also most directly relevant to implementation in Canadian clinical trial settings. However, we acknowledge that this restriction may introduce language bias and may exclude relevant resources developed in other linguistic or regional contexts. This will be noted as a limitation of the review.

### Study selection process

Two independent reviewers will screen titles and abstracts using the predefined eligibility criteria. Records that appear eligible or require further assessment will proceed to full-text review. Two reviewers will independently assess full-text articles and resources for inclusion. Disagreements at any stage will be resolved through discussion or consultation with a third reviewer.

Reasons for exclusion at the full-text stage will be documented. The study selection process will be summarized using a PRISMA flow diagram.

### Data extraction

Data will be extracted using a standardized and pre-piloted data extraction form developed in Covidence. The form will be piloted by the review team on a sample of included resources and refined as needed to ensure consistency and completeness.

Extracted data will include:

Toolkit identification and background information: title, author or organization, year of publication, country or region of origin, type of resource, intended users, and access link.
Purpose and scope: stated objective, target audience, intended setting, and clinical trial phase addressed.
IDEA domains addressed: inclusivity, diversity, equity, accessibility, or combinations of these domains.
Target populations: women, Indigenous population, racialized communities, immigrants and refugees, rural and remote populations, people with disabilities, older adults, sexual and gender minorities, people with lower socioeconomic status, and other underrepresented or underserved groups.
Practical components: checklists, templates, frameworks, training modules, assessment tools, implementation guides, examples, case studies, decision aids, recruitment or retention strategies, and dissemination tools.
Usability features: clarity of instructions, organization of content, availability of step-by-step guidance, user-friendly format, examples or case studies, implementation supports, and adaptability across settings.
Evidence base and development process: whether the toolkit describes its evidence base, development methods, stakeholder involvement, pilot testing, evaluation, or revision process.
Applicability to the Canadian context: relevance to the Indigenous population, bilingual considerations, multicultural communities, rural and remote populations, people with disabilities, Canadian legal or regulatory frameworks, and Canadian clinical trial infrastructure.

Any disagreements during data extraction will be resolved through discussion among reviewers or consultation with a third reviewer.

#### Descriptive appraisal of toolkit robustness

Although formal critical appraisal is not typically required for scoping reviews, this review will include a descriptive appraisal of the robustness and practical utility of included toolkits. This appraisal will not be used to exclude resources. Instead, it will help characterize the strength, transparency, and potential usefulness of each toolkit.

The descriptive appraisal will assess whether each toolkit reports:

1. A clear purpose and intended audience;
2. A stated evidence base or supporting literature;
3. A transparent development process;
4. Stakeholder, patient, community, or end-user involvement;
5. Pilot testing, evaluation, or feedback from intended users;
6. Practical implementation guidance;
7. Accessibility features;
8. Adaptability across trial settings or populations;
9. Relevance to Canadian clinical trial practice.

Findings from this appraisal will be summarized descriptively and presented in tabular form to support comparison across included toolkits.

#### Data analysis and synthesis

Findings will be synthesized descriptively using tables, narrative summaries, and comparative matrices. Included toolkits will be mapped according to their purpose, intended users, IDEA domains addressed, clinical trial phase addressed, target populations, practical components, usability features, evidence base, and applicability to the Canadian context.

We will use predefined categories to compare toolkits across the following dimensions:

1. Toolkit characteristics
2. IDEA domains addressed
3. Clinical trial phases addressed
4. Target populations and equity-relevant groups
5. Practical tools and implementation support
6. Usability indicators
7. Evidence-based and development process
8. Canadian applicability

The synthesis will identify common components across toolkits, gaps in content or populations addressed, and examples of promising practices that may inform the development or adaptation of future IDEA toolkits for clinical trials in Canada (refer to Appendix 2 for specific details).

## Discussion

This scoping review will identify and describe existing toolkits designed to support IDEA in clinical trials. By mapping toolkit content, intended users, practical components, target populations, usability features, evidence base, and Canadian applicability, this review will provide a structured overview of available resources and their relevance to inclusive clinical trial practice.

The findings will be particularly important for the Canadian clinical trial context. Canada’s population is diverse with respect to geography, language, culture, race, ethnicity, socioeconomic status, disability, immigration status, and Indigenous identity. Clinical trial participation may be shaped by barriers related to geography, transportation, language, mistrust, cultural safety, disability access, digital access, and health system navigation. Therefore, toolkits designed for inclusive clinical trials must be assessed not only for their general content but also for their practical usability and relevance to Canadian settings.

This review will help identify which toolkit components are commonly available and which areas remain underdeveloped. For example, some toolkits may focus primarily on recruitment but provide limited guidance on retention, consent, accessible communication, trial conduct, disaggregated analysis, or dissemination to participants and communities. Others may provide broad equity principles but lack practical tools such as templates, checklists, training materials, or implementation guidance. By examining these features systematically, the review will help identify promising practices and gaps that can inform future toolkit development.

Given the applied focus of this review, findings will be shared with relevant stakeholders, including clinical trial units, researchers, patient partners, and equity-focused groups, to inform future toolkit development and adaptation. We recognize that direct involvement of patients, Indigenous communities, and other underrepresented groups in the conduct of this scoping review may be limited. This will be acknowledged as a limitation. Future work should include meaningful engagement with these groups to ensure that toolkit recommendations are grounded in lived experience, community priorities, and practical implementation needs.

This review has several limitations. First, limiting included resources to English and French may exclude relevant toolkits developed in other languages or regions. Second, because toolkit-related terminology is broad and inconsistently used, some relevant resources may not be captured despite a comprehensive search strategy. Third, included resources are expected to vary substantially in format, scope, evidence base, and level of implementation guidance, which may limit direct comparison across toolkits. Finally, although the review will assess whether toolkits report stakeholder involvement, direct patient, Indigenous, or community engagement in the review process itself may be limited. These limitations will be considered when interpreting the findings and developing recommendations for future toolkit adaptation.

## Ethics statement

Ethics approval is not required for this scoping review because it will use publicly available literature and resources. The findings from this review will be disseminated through peer-reviewed publications, conference presentations, and stakeholder engagement activities.

## Footnotes

1
Embase.com

## Group members of ACT AEC Consortium Leadership and IDEA Subcommittee

Accelerating Clinical Trials (ACT) consortium leadership Drs. PJ Devereaux, Guy Rouleau, and Inclusivity, Diversity, Equity, and Accessibility Committee members- Diego Herrera, PhD., Leanne Casaubon, PhD., David Collister, MD, PhD., Sarina Isenberg, Alexandra King, Patricia Li, MD., FRCPC., Heather Lochnan, MD., Courtney Pollock, PhD., Amity Quinn, PhD., Shannon Ruyzcki, PhD., Shelley Zieroth, PhD. Accelerating Clinical Trials Consortium, Hamilton, ON, Canada.

## References

[1] Alsan M Wanamaker M. Tuskegee and the health of black men. Q J Econ. (2018) 133:407–55. doi: 10.1093/qje/qjx029, 30505005 PMC6258045

[2] Ruzycki SM Ahmed SB. Equity, diversity and inclusion are foundational research skills. Nat Hum Behav. (2022) 6:910–2. doi: 10.1038/s41562-022-01406-7, 35768648 PMC9243902

[3] Garcia M Mulvagh SL Merz CN Buring JE Manson JE. Cardiovascular disease in women: clinical perspectives. Circ Res. (2016) 118:1273–93. doi: 10.1161/circresaha.116.307547, 27081110 PMC4834856

[4] Ramamoorthy A Pacanowski MA Bull J Zhang L. Racial/ethnic differences in drug disposition and response: review of recently approved drugs. Clin Pharmacol Ther. (2015) 97:263–73. doi: 10.1002/cpt.61, 25669658

[5] Clark LT Watkins L Piña IL Elmer M Akinboboye O Gorham M . Increasing diversity in clinical trials: overcoming critical barriers. Curr Probl Cardiol. (2019) 44:148–72. doi: 10.1016/j.cpcardiol.2018.11.002, 30545650

[6] Sirugo G Williams SM Tishkoff SA. The missing diversity in human genetic studies. Cell. (2019) 177:26–31. doi: 10.1016/j.cell.2019.02.048, 30901543 PMC7380073

[7] Goldman DP Cutler D Rowe JW Michaud PC Sullivan J Peneva D . Substantial health and economic returns from delayed aging may warrant a new focus for medical research. Health Aff. (2013) 32:1698–705. doi: 10.1377/hlthaff.2013.0052, 24101058 PMC3938188

[8] Giesbrecht MD Crooks VA Morgan J. "Place, health, and diversity in Canada". In: Giesbrecht M and Crooks V, editors. Place, Health, and Diversity. Ashgate Publishers. (2016). p. 1–13.

[9] Tricco AC Lillie E Zarin W O'Brien KK Colquhoun H Levac D . PRISMA extension for scoping reviews (PRISMA-ScR): checklist and explanation. Ann Intern Med. (2018) 169:467–73. doi: 10.7326/M18-0850, 30178033

[10] Foster ED Deardorff A. Open science framework (OSF). J Med Libr Assoc. (2017) 105:203. doi: 10.5195/jmla.2017.88

[11] Dykes PC Duckworth M Cunningham S Dubois S Driscoll M Feliciano Z . Pilot testing fall TIPS (tailoring interventions for patient safety): a patient-centered fall prevention toolkit. Jt Comm J Qual Patient Saf. (2017) 43:403–13. doi: 10.1016/j.jcjq.2017.05.002 28738986

[12] National Institute of Health Research. The INCLUDE Ethnicity Framework. (2020).

