# Supplementary material for: Evaluating toolkits for inclusivity, diversity, equity, and accessibility in clinical trials: a scoping review protocol
Source: Front Public Health. 2026 Jun 22;14:1824000. doi: 10.3389/fpubh.2026.1824000 (PMC13335071; doi:10.3389/fpubh.2026.1824000)
Supplement: Supplementary file 1 [file Data_Sheet_1.pdf]

Appendix 1
Search Criteria

Ovid MEDLINE(R) Epub Ahead of Print and In-Process, In-Data-Review & Other Non-Indexed Citations and Daily <July 01, 2024>

1 "health disparate minority and vulnerable populations"/ or "sexual and gender minorities"/ or vulnerable populations/ 25943

2 "ethnic and racial minorities"/ 742

3 Minority Groups/ 18838

4 Cultural Diversity/ 13214

5 Diversity, Equity, Inclusion/ 391

6 (equity or diversity or EDI or EDI or DEIA or BIPOC or POC or "people of colo?r" or "women of colo?r" or "men of colo?r" or BAME).tw,kf. 396529

7 ((underserved or minority or underrepresented or under served or under represented or marginalized or vulnerable) adj1 (group* or population* or communit*)).tw,kf. 56797

8 1 or 2 or 3 or 4 or 5 or 6 or 7 488110

9 exp Clinical Trials as Topic/ 394002

10 (clinical adj1 (trial* or research or study or studies)).tw,kf. 759166

11 (controlled trial or RCT or (random* adj2 trial*)).tw,kf. 512719

12 9 or 10 or 11 1328699

13 exp Guidelines as Topic/ 174287

14 exp Guideline/ 38809

15 (toolkit* or framework* or guideline* or guidance or tool or tools).tw,kf. 2071292

16 13 or 14 or 15 2173373

17 8 and 12 and 16 2759

Appendix 2

Key Components of the Data Extraction Form

PREVIEW

1. Study/Article identification

Table preview

Study Id	Study Title	Authors	Year Published

Notes

2. Toolkit Identification and Background Information

Table preview

Toolkit Title/link	Organization	Year Published	Intended audience (e.g., researchers, healthcare providers, policymakers)	Geographic origin/country

Notes

3. Content and Purpose

Table preview

Toolkit objective (e.g., enhancing recruitment diversity, promoting equity)	Guiding principle (e.g., cultural competence, or OCAP principles)	Focus Areas (e.g., inclusion, accessibility, equity)	Phase of the trial addressed (e.g., Recruitment phase, trial execution phase, Analysis of results, and Dissemination of findings)

Notes

4. Key Components

1. Guidance and framework
2. Training modules
3. templates/Checklists
4. Tools for IDEA
5. case studies or real-time examples
6. others

Notes

5. Applicability to the Canadian Context? Yes/No

Yes/No

1. Yes
2. No

If Yes, check if the toolkit has following:

1. Bilingual Content
2. Indigenous Considerations
3. Population Focus (e.g., immigrants, refugees, rural/remote populations)
4. Align with Canadian laws (e.g., CIHR, Health Canada)

Notes
